# Impact of altitudinal gradients on biochemical traits and fatty acid profiles of Iranian hazelnuts

**DOI:** 10.1186/s12870-025-07750-w

**Published:** 2025-12-01

**Authors:** Navid Rezvanjo, Alireza Ghanbari, Asghar Estaji, Amir Mohammad Naji, Hassan Maleki Lajayer, Hashem Kazemzadeh-Beneh

**Affiliations:** 1 Department of Horticultural Sciences, University of Mohaghegh Ardabili, Ardabil, Iran; 2 Sugar Beet Seed Institute (SBSI), Agricultural Research Education and Extension Organization (AREEO), Ardabil, Iran; 3 Department of agronomy, Faculty of Agricultural Science, Shahed University, Tehran, Iran; 4 Faculty of Agriculture (MeshkinShahr Campus), University of Mohaghegh Ardabili, MeshkinShahr, Iran; 5 Division of Biotechnology & Plant Molecular Genetic, Department of Horticulture Science, University of Hormozgan, Bandar Abbas, Iran

**Keywords:** Altitudinal gradients, Antioxidants capacity, Fatty acids, Flavonoid, Phenolic, Hazelnuts

## Abstract

This study investigates the influence of altitudinal gradients on the fatty acid composition, antioxidant capacity, flavonoid and phenolic content of eight hazelnut cultivars. Samples were collected from orchards at low (0–500 m), mid (500–1000 m), and high (> 1000 m) altitudes in Gilan province of Iran. Duncan’s test (*p* ≤ 0.01) revealed that altitudinal gradients were significantly alternated the antioxidant capacity, phenolic, and flavonoid contents of hazelnut cultivars. With increasing altitude, a decreasing trend in the antioxidant capacity (ranged from 74.33 to 27.33%) and phenolic content (ranged from 380.22 to 104.68 mg GAE/ 100 g dw) was observed. In contrast, a fluctuating trend in flavonoid content was ranged from 60.33 to 170.99 (mg QE/g dw) across altitudinal gradients; subsequently Round cultivar showed the highest flavonoid at mid altitude. In terms of fatty acids, an increase in the biosynthesis of unsaturated acids, including oleic (46.93–82.37%) and linoleic acid (0.1–6.52%) occurred at high altitude. While an increase in the concentration of saturated acids such as palmitic acid (3.61–37.86%), behenic acid (0.11–0.55%), stearic acid (3.12–7.5%), arachidic acid (0.41–6.05%), 11-Eicosenoic acid (0.33–6.19%), and myristic acid (0.11–0.69%) happened at low altitude. It was found that the ratio of unsaturated/saturated acids increased along with rising altitude, and vice versa. Conversely, the production of palmitoleic, as unsaturated acids, and tetracosanoic and pentadecanoic acids, as saturated acids, induced at low and high altitudes, respectively. In contrast, under all three altitudes condition, the biosynthesis of the oxirane octanoic was enhanced remarkably. These results highlight that the alterations in biochemical and fatty acid profile of hazelnut were cultivar type-, altitude-, and altitude-cultivar type interaction dependents. This aspect validated by PCA and heatmap analysis, which segregated cultivars into distinct groups. This highlights the importance of environmental and genetic factors in shaping hazelnut quality. These findings underscore the significance of considering altitude and cultivar selection in hazelnut cultivation to optimize nutritional quality, which can be valuable to producers, breeders, and the food industry.

**Supplementary Information:**

The online version contains supplementary material available at 10.1186/s12870-025-07750-w.

## Introduction

Iran country, with an annual production of 15,000 to 20,000 metric tons, has been ranked consistently among the world’s top ten producers of hazelnut fruit. This represents about 1.5% of total world hazelnut production that steady presence in international nut markets [1]. The story of Iranian hazelnuts is fundamentally tied to geography. Production concentrates heavily along the Khazar Sea coastline, where the provinces of Gilan and Mazandaran account for over 80% of national output. Within these regions, districts like Rudsar, Amlash, and Rezvanshahr have developed specialized expertise, becoming veritable hubs of hazelnut cultivation and processing. Gilan province produces 85% of Iran’s hazelnuts, Eshkevarat, and Rahimabad in Rudsar city are the largest hazelnut producers and are known as the hazelnut capital of Iran. Gillan’s success with hazelnuts stems from perfect growing conditions. The region enjoys a unique humid subtropical climate with annual rainfall between 1,000 and 1,200 mm [2]. The acidic, fertile soils of the foothills create ideal rooting conditions, while genetic diversity among local varieties provides natural resilience [2].

The hazelnut (*Corylus avellana* L.) from Betulaceae family is categorized as a rich source of nutritional value and health-boosting functional food due to the presence different compounds in its nut fruit such as phenolic compounds, fatty acids (monounsaturated and polyunsaturated fatty acids), lipids, vitamins E, and antioxidant compounds ‎ [3]. Owing to these beneficial compounds, hazelnuts mainly contribute to the multiple therapeutic effects, including anti-inflammatory, cardiovascular protection, and antioxidant activities ‎ [3–5]. The lipid composition of hazelnuts is notable for several fatty acids including oleic, linoleic, palmitic, stearic, and arachidic acids [6]. Oleic acid is the predominant fatty acid, followed by linoleic, palmitic, and stearic acids‎ []. Linoleic acid is associated with reduced atherosclerosis, while oleic acid helps lower blood cholesterol. Linoleic and linolenic acids contribute to blood pressure regulation by decreasing blood lipid and glycerol levels ‎ [7]. Hazelnuts are also mineral-rich, containing approximately 1–3.4.4% ash. A 100-gram serving can provide the daily mineral requirements for magnesium, iron, potassium, manganese, copper, phosphorus, zinc, and calcium ‎ [8, 9]. Furthermore, the high antioxidant capacity of the hazelnuts is associated with the flavonoids and antioxidants like catechin, epigallocatechin gallate, proanthocyanidins, dimeric procyanidin B2, and quercetin 3-rhamnoside, and tocopherols ‎ [9–11]. It is notable that numerous aspects, including variety, climate conditions, location, agricultural practices, and microbial contamination, can influence or decrease the chemical composition of hazelnut fruits ‎ [12, 13]. Therefore, the biochemical profile of hazelnut fruits is manipulated by a complex interaction of genetic background and environmental factors.

Under environmentally stressful conditions, the response rate of plants to physiological and biochemical changes manifests through phenotypic plasticity and alterations in morpho-physiological and phytochemical approaches ‎[‎^14^, ^16^]. Correspondingly, environmental factors such as drought, salt, cold, heat, ultraviolet light (UV-B ultraviolet irradiation), and altitude can fundamentally cause variations in the generation of secondary compounds among plants, depending on their genetic background ‎ [17–19]. In this regard, plants residing along high-altitudinal gradients are exposed to undesirable environmental stress conditions including dehydration, low oxygen levels, high UV irradiation, low temperatures, frozen soils in winter, atmospheric pressure decreases, and weathering ‎ [20]. Nevertheless, earlier studies have exhibited that plants overcome to the undesirable situations of altitudinal gradients by stimulating the synthesis of phenolics, alkaloids, flavonoids, tannins, lipids, and terpenoids, which regulate the plant acclimatization to different environmental conditions ‎ [21, 22]. Therefore, to date, many investigations have clearly been confirmed that there is a positive relationship between the high levels of antioxidants, phenolics, carotenoids, and secondary metabolite levels and the plants adapted to climatic conditions at a high altitude ‎ [14, 15, 23, 24]. Under this status, the ability of one plant to synthesize secondary metabolites will differ considerably from another plant due to differences in their genetic background ‎ [16, 25–27]. It has already been proven that the synthesis level of primary metabolites in plant tissues declines under high altitudinal gradients, while the synthesis level of secondary metabolites enhances significantly ‎ [23]. In this sense, the stimuli of high-altitude gradients on the synthesis of flavonoids and antioxidant compounds in some endemic plants ‎ [14], phenols and valerenic acid in *Valeriana jatamansi* ‎ [28], carotenoid content in peach ‎ [29], the accumulation of vanillic acid, ferulic acid, and caffeic acid in *Angelica sinensis* ‎ [30], and antioxidants and antimicrobial properties ‎ [31] have previously been stated.

The altitudinal gradient has appeared as a fundamental environmental factor that powerfully induces secondary metabolites, the synthesis of biochemical profiles, and fatty acid compositions in hazelnut fruits ‎ [32–34]. In the literature, recent studies have exclusively explored the environmental variation of fatty acid profiles, phenolic, flavonoid, and antioxidant compounds in the Turkish and Italian hazelnut fruits under high-altitude environments ‎ [11, 32, 40]. They highlighted that the fatty acid compositions, phenolic, flavonoid, and antioxidant compounds positively enhanced in fruits by increasing the altitude in hazelnut growing areas. This indicates the interaction of genetic and environmental factors in shaping the chemical composition profile of hazelnuts. Likewise, these studies confirmed that the different cultivars of hazelnuts grown under similar altitudinal gradients revealed different potentials in fatty acid composition, tocopherol content, and phenolic compounds, as has been confirmed by Cristofori et al. ‎ [34]. This emphasizes the importance of the genetic background of different hazelnut cultivars in the synthesis of biochemical compounds. Therefore, these conflicting results suggest that the influences of altitude may be cultivar-specific or dependent on other environmental factors, which are determined by the prevailing climate conditions of hazelnut growing areas ‎ [35–39]. Hence, there is a prominent gap in knowledge associated with the effect that the altitude gradient may have on fatty acid compositions, phenolic, flavonoid, and antioxidant compounds in Iranian hazelnut cultivars and thus, their genetic-altitude gradient interaction remains poorly understood. Accordingly, the current study intends to elucidate this gap by exploring the effect of altitude gradient on the fatty acid composition profile as well as the flavonoid, phenolic and antioxidant capacity of eight commonly hazelnut cultivars grown at different altitudes under diverse climatic conditions in the northern region of Iran country. This work has basic implications for future research to provide valuable insight for improving hazelnut cultivation practices in diverse agro-ecological areas and to meet the growing demand for functional foods.

## Materials and methods

### Experimental sites and cultivar sampling

**Fig. 1:**
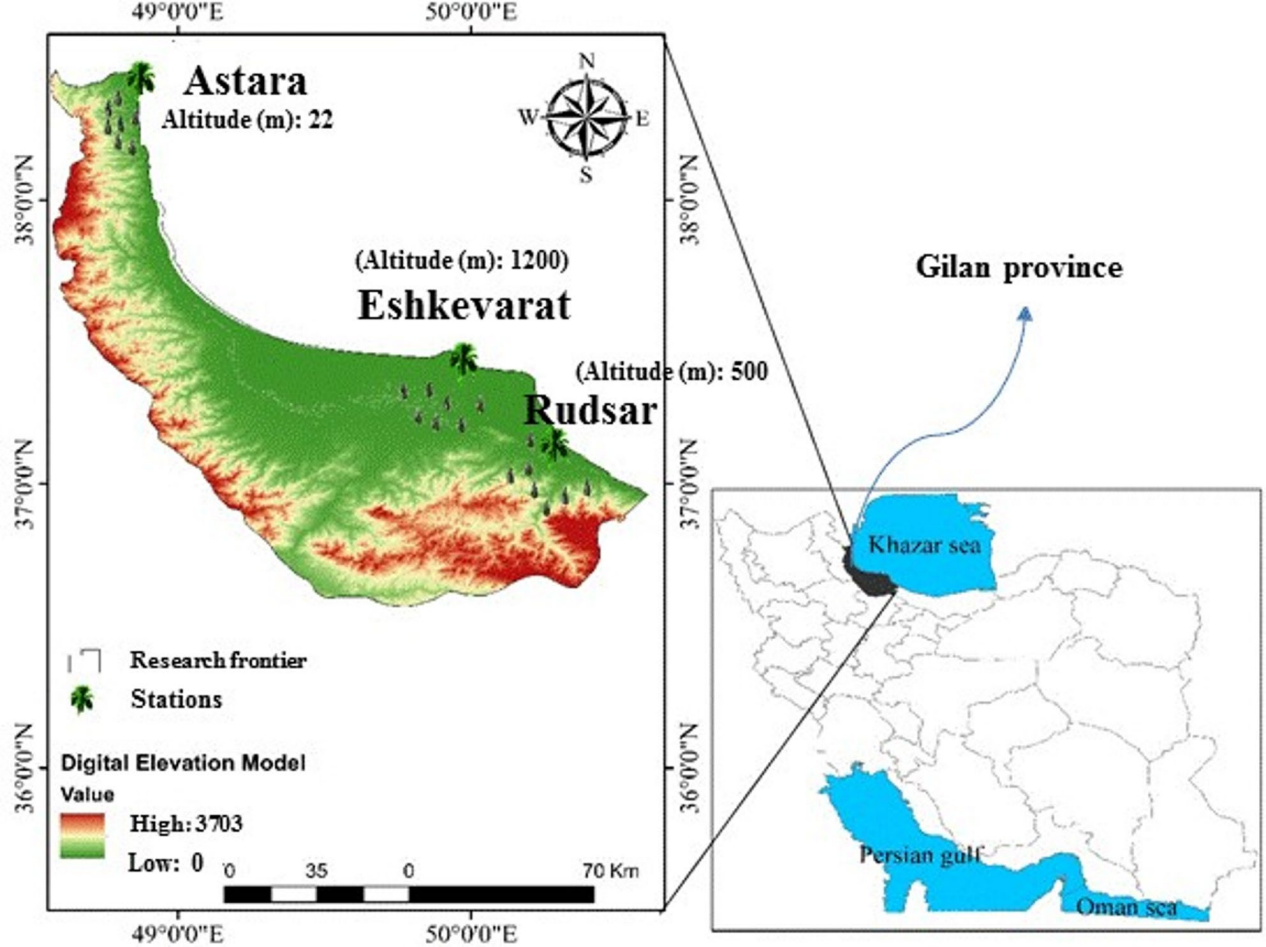
Map of the study area showing the sampling locations from three regions of Gilan province (Astara, Rudsar, and Eshkevarat), Iran

This study was performed in three regions of Gilan province including Astara, Rudsar, and Eshkevarat cities, in the northern region of Iran during the year 2024 (The study was only conducted a single year in 2024), with spatial specifications and map of the location sampling as revealed in Table 1; Fig. 1, respectively. The Gilan province is situated in the western Khazar Sea of Iran. The climatic data of sampling sites in Gilan province, including high and low temperature, average monthly rainfall, and humidity comfort level were detailed in the Supplementary Fig. S1, Fig. S2, and Fig. S3. The factorial completely randomized design (CRD) as experimental design was conducted, where each experimental unit was linked to 16 m^2^ (4 m × 4 m) with a 3 × 8 factorial combination. One factor corresponded to the three altitudinal gradients low (0–500 m), mid (500–1000 m) and high (> 1000 m)), while another factor corresponded to the eight hazelnut cultivars assessed (Ganjeh, Doroucheh, Fertile, Round, Atrak, Namsa, Segorb and Kouban), each with three replications (i.e., for a total of 24 treatments). In the mentioned regions, healthy hazelnut trees with no obvious symptoms of biotic and abiotic stresses, insect damage, or other issues were randomly chosen for sampling. Gardening practices including pruning, irrigation, fertilization, and plant protection are regularly monitored under same conditions in the orchards. The same values of pH (5.3–6.5) and soil type (sandy loam) were observed in three regions. Fruits were harvested at the equal maturity stage (when the green shells changed yellow, and the moisture content fell to 30%) from three regions. Fruits of each cultivar, homogeneous size, same color, exposure and agronomic conditions, were chosen across the three altitudinal gradients. The fruit samples were obtained in September in 2024 from orchards located at three altitudinal gradients in Gilan province of Iran (Rudsar County). For fatty acid profiling and biochemical assays, the nuts of the cultivars were unshelled and handled immediately to inhibit quick degradation of bioactive compounds. Subsequently, their kernels were milled and stored in polyethylene bags at −20 °C.

**Table 1:**
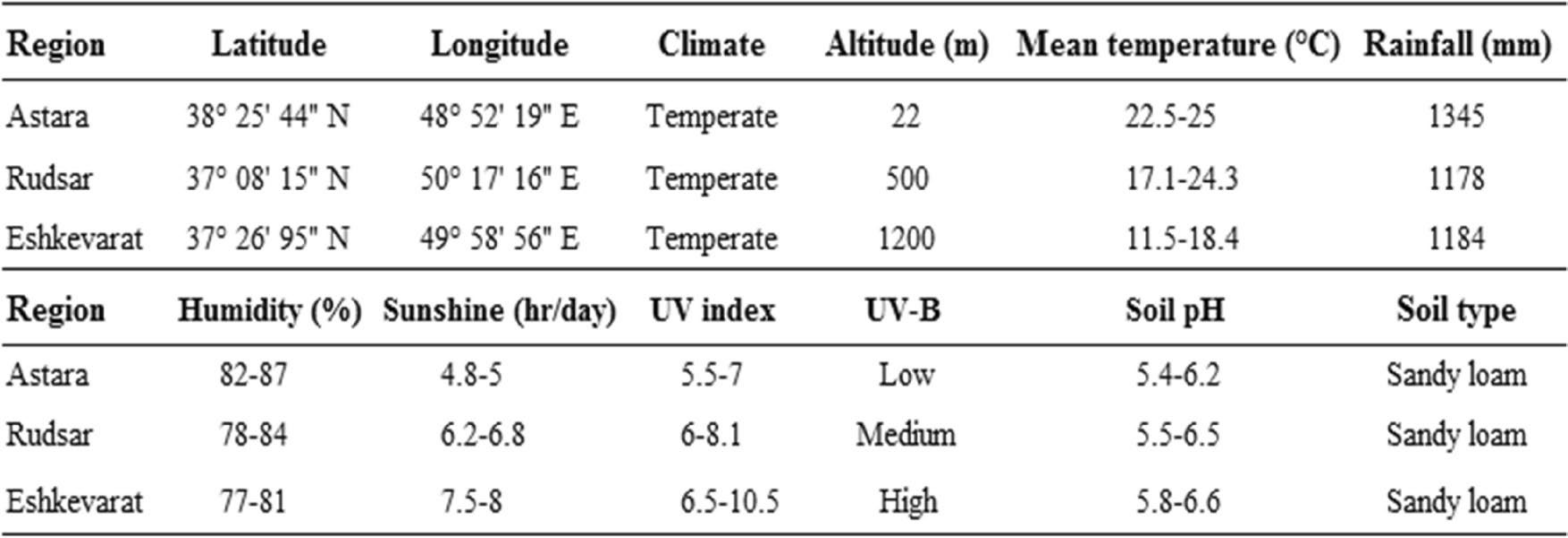
Location and geographical position, and Climatic data of the experimental regions of Gilan province, Iran

### GC-MS analysis for fatty acids characterization

The total oil in the hazelnut kernels was extracted using a Soxhlet apparatus, with the inclusion of light petroleum ether (boiling point 40–60 °C). About 9 g of each sample were ground, powdered, and placed on filter paper. The content was extracted with approximately 200 mL of petroleum ether for 6 h using by the Soxhlet apparatus. In the next step, the solvent was left to evaporate, and the remaining oil was obtained [22]. The fatty acid composition of hazelnuts was determined by a gas chromatography device (GC-MS: Gas Chromatography-Mass Spectrometry), which assessed the types of fatty acids and their proportions through methyl derivatives. The American Oil Chemist Society (AOCS) Ce 1b-89 protocol was applied for analyzing the composition of the hazelnut oil fatty acids ‎[‎ 41]. Accordingly, a 0.5 mL solution of tridecanoic acid, with a concentration of 0.5 g/L, was added in the vials equipped with screw caps (containing 0.09 g of oil). At that point, 2 mL of methanolic potassium hydroxide 0.5 M was added, and the vials were warmed for 30 min at 60 °C. After cooling the sample, 2 mL of 10% BF_3_ in methanol was supplemented, and the sample was maintained once more at 60 °C for 30 min. This was followed by cooling the mixture, adding 2 mL of hexane, and shaking the mixture for one min. The 2 mL of a saturated solution of fluid sodium chloride was added after mixing. The solution was centrifuged, and the hexane layer was transferred to a vial for analysis in the GC-MS device. The composition of fatty acid methyl esters was analyzed via gas chromatography. N_2_ was used as a carrier gas at a flow rate of 1 mL/min. For the separation of the fatty acids, the temperatures of the column, detector, and injection were set at 240 °C ‎[‎ 42]. The outcome of analysis was reported as a percentage of fatty acids, according to the software that worked in tandem with the GC-MS device. Tridecanoic acid (C13:0) was considered the internal standard for the GC-MS analysis.

### Antioxidant activity assay

The assay of DPPH (2,2-diphenyl-1-picrylhydrazyl) radical scavenging activity was considered as the antioxidant activity (which was defined by measuring the ability of antioxidants to reduce DPPH· radical) of hazelnut fruit extracts according to the method previously described by Brand-Williams et al. ‎[‎ 43]. The kernels of hazelnuts were powdered and subsequently, was subjected to extraction by centrifuging at 5000 rpm for 5 min. A 300 µL of prepared solution of 0.004% DPPH was introduced into a mixture tube containing 30 µL aliquots of each extract and 2650 µL of methanol and then was incubated in the dark condition for 30 min after thorough mixing. Ultimately, the optical absorption of the achieved mixture was recorded at 517 nm using a UV-Vis’s spectrophotometer (Agilent Cary-60, Santa Clara, CA, USA). The formula for DPPH calculation (DPPH radical scavenging capacity (%) = (A control – A sample) × 100/A control; where A sample and A control represent the absorbance of the sample absorbance of the control, respectively) was utilized for antioxidant activity assay. In this regard, the inhibition concentration (IC_50_) values for reporting the results of antioxidant activity were established by plotting the inhibition ratios versus the regression line attained from eight different sample concentrations and applying the regression equation ‎[‎ 44].

### Total phenolic content (TPC) evaluation

The Folin–Ciocalteu spectrophotometric approach was applied to TPC content evaluation of hazelnut kernels as previously described by Waterhouse. ‎[‎ 45]. In this regard, a mixture solution (3 mL volume) in a tube containing 50 µL of kernel extract solution, 250 µL of Folin–Ciocalteu reagent, and 2.7 mL of distilled water was prepared in the initial step and then was incubated at room temperature for 5 min. In the next step, 750 µL of 20% (w/v) Na_2_CO_3_ solution was introduced into the mixture solution in tube and then was mixed in a vortex. The obtained mixture was maintained in the dark condition at room temperature for 90 min. In the last step, the absorbance of the resulting mixtures related to each hazelnut kernel was recorded at 765 nm in a UV-Vis spectrophotometer (Agilent Cary-60, Santa Clara, CA, USA) against the distilled water as a blank serving as the reference. To generate the standard curve of gallic acid for TPC calculation, a similar procedure was conducted to prepare a serial dilution of gallic acid solution with concentrations of 50, 100, 150, 200, and 300 µg/mL. The TPC content of each hazelnut kernel was reported as gallic acid equivalent (mg GAE/100 g nuts dry weight).

### Total flavonoid content (TFC) assay

TFC of hazelnut kernels was predicted colorimetrically by applying aluminum chloride according to protocol suggested by Zao et al. ‎[‎ 46]. One mL of the extracted hazelnut kernel in methanol was suspended in 4 mL of distilled water. Then, 1 mL of 5% sodium nitrite was introduced into obtained mixture and was incubated at room temperature for 5 min. Subsequently, 1 mL of 10% aluminum chloride and 8 mL of sodium hydroxide were included in the mixture, respectively. In this assay, an orange yellowish color appears due to interaction between the aluminum chloride and hydroxyl groups of flavonoids present in the kernel extract. The absorbance of colored solution was spectrophotometrically measured at 510 nm against blank serving as reference. The blank solution was considered to be a mixture solution containing all reagents except for the kernel extract. A standard curve of quercetin was applied to TFC prediction and thus, the results were expressed as quercetin equivalents (QE) per milligram of dry weight of hazelnut kernel (mg QE/g dw).

### Statistical analysis

The study was conducted using a factorial completely randomized design (CRD) with two main factors including altitudinal gradients (with three levels) and cultivar (with eight levels), which was considered three replications for each level. The Kolmogorov-Smirnov test was applied to validate the normal distribution of variables. Statistical differences were computed using two-way analysis of variance (ANOVA) to determine the differences in means between the impacts of different hazelnut cultivars and their interaction with the altitude gradients on the measured traits. Data were analyzed by using XLSTAT software (version 2014.5). Mean comparisons were performed based on Duncan’s New Multiple Range Test (DNMRT) at *p* ≤ 0.01. Principal component analysis (PCA) and heatmap cluster analysis based on Euclidean distance were performed to detect patterns and relationships among the variables.

## Results

Generally, the ANOVA variance analysis showed that the altitudinal gradients, cultivar types, and their interaction had significant effect (*p* ≤ 0.01) on biochemical traits and fatty acid (FA) composition of hazelnut fruits (Supplementary Table S1 and Table S2).

### The impact of altitude gradients and cultivar types on antioxidant activity

**Fig. 2:**
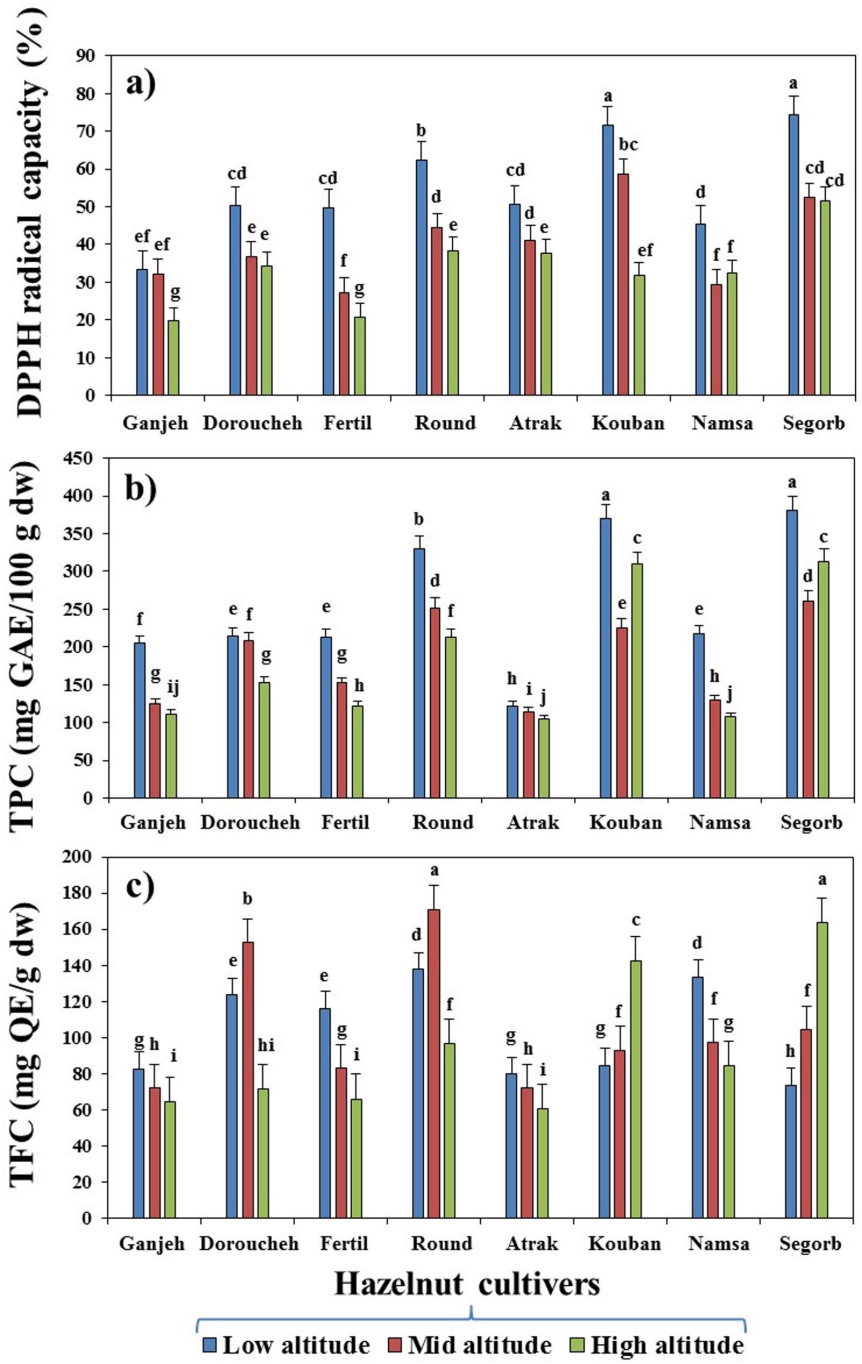
DPPH radical scavenging capacity (**a**), total phenolic contents (**b**), total flavonoid of kernel’s hazelnut cultivars according to the altitudinal gradients (low altitude: 0–500 m; mid altitude: 500–1000 m; high altitude: >1000 m). Difference letters at the top of the columns exhibit significant difference for hazelnut cultivars-altitude interaction based on Duncan’s New Multiple Range Test (DNMRT) at *p* ≤ 0.01

As per the Duncan’s mean comparison test, the significant variation in the antioxidant activity among the hazelnut cultivars was explored under altitudinal gradients. The antioxidant activity (DPPH radical scavenging capacity) of hazelnut’s kernels varied greatly depending on the altitude gradient, cultivar type, and their interaction (Fig. 2a).

Indeed, the DPPH technique assesses the competence of antioxidant compounds present in hazelnut fruit extracts to scavenge DPPH· radicals. Overall, the antioxidant activity of hazelnut cultivars revealed a sharp reduction with the rise in altitudinal gradients from 22 to 1000 m; however, each cultivar exhibited the heightened DPPH activity capacity at lower elevations (22 m) compared to higher elevations (> 500 m). Generally, the antioxidant activity of cultivars varied from 33.33 to 74.33% at low altitude (0–500 m) and 19.67 to 51.67% at both mid and higher altitude gradients (500–1200 m). This confirms that antioxidant activity is not only affected by low altitude, but also by the type of cultivar. In this regard, Segrob, Kouban, and Round cultivars flaunted the topmost DPPH inhibition percentages, with means of 74.33, 71.67 and 62.33%, respectively, whereas the Ganjeh cultivar revealed the bottommost antioxidant activity, with a mean of 19.67% for DPPH inhibition percentage. Likewise, the superior antioxidant constitution of Segrob and Kouban cultivars was observed remarkably at all altitude gradients, while the other cultivars demonstrated a similar potential antioxidant power with slightly different variations at altitudes more than 500 m, depending on cultivar type (Fig. 2a). This indicates that the admirable antioxidant power of Segrob and Kouban cultivars can be associated with their superior genetic background, which is potentially affected by altitudinal gradients.

###  The TPC and TFC variations in hazelnuts cultivar under altitude gradients

The obtained results based on Duncan’s test (*p* ≤ 0.01) obviously evidenced that fluctuations in the altitudinal gradients resulted in a significant alteration in the content of TPC and TFC secondary metabolites in hazelnut fruits, relying on potential genetic background of the investigated cultivars (Fig. 2b and c). Therefore, the cultivar type-altitude gradient interaction plays a main role in generating the target secondary metabolites. The highest TPC was attained at low altitude; subsequently, the generation of TPC declined with the rising altitudinal gradients beyond 1000 m in the kernels of hazelnut cultivars. The TPC compound expanded from a 107.33 mg GAE/100 g dry weight at higher altitude reached to a 380.22 mg GAE/100 g dry weight at the lower altitude. Similar to the antioxidant activity, the low-altitude cultivars (including Segorb (380.22 mg GAE/g dw), Kouban (369.67 mg GAE/100 g dw), and Round (330.68 mg GAE/100 g dw), respectively) not only exhibited the most potent in TPC production but were also the superior cultivars across all altitudinal gradients for TPC production compared to other cultivars (Fig. 2b). Moreover, considering the individual cultivars, it was detected that the higher altitude led to a notable reduction in TPC content in all cultivars except for Segorb and Kouban. Despite the reduction in TPC content in the Segorb and Kouban cultivars sampled from low altitude, the TPC obtained from them was still higher compared to other cultivars. Consequently, the lowest level of TPC belonged to Namsa with a mean of 107.33 mg GAE/100 g dw and Ganjeh with a mean of 111.67 mg GAE/100 g dw, respectively.

Similar to TPC variation in hazelnut cultivars based on altitudinal gradients, the sampled cultivars indicated a different behavior for TFC production under varied altitudinal gradients, ranging from 84.33 to 170.99 mg QE/g dw (Fig. 2c). The TFC production in kernels of Ganjeh, Fertil, Atrak, and Namsa cultivars decreased gradually with rising altitude, while the Segorb and Kouban cultivars revealed a notable increase in TFC content along with the rise in altitude. On the conflicting side, the Round (with TFC level of 170.99 mg QE/g dw) and Doroucheh (with TFC level of 152.87 mg QE/g dw) cultivars sampled from mid altitude presented the best performance for TFC production in their kernels, respectively. This is suggesting that the power for TFC production in hazelnut cultivars’ kernels is intensively affected by the altitudinal gradients and cultivar type interaction more than their capability for TPC and antioxidant activity. Parenthetically, the Segorb and Kouban sampled from high altitude, the Round and Doroucheh sampled from mid altitude, and Segorb and Kouban sampled from low altitude introduced as the superior cultivars for TFC production.

### GC-MS characterization of fatty acid composition of hazelnut cultivars under altitude gradient

**Fig. 3:**
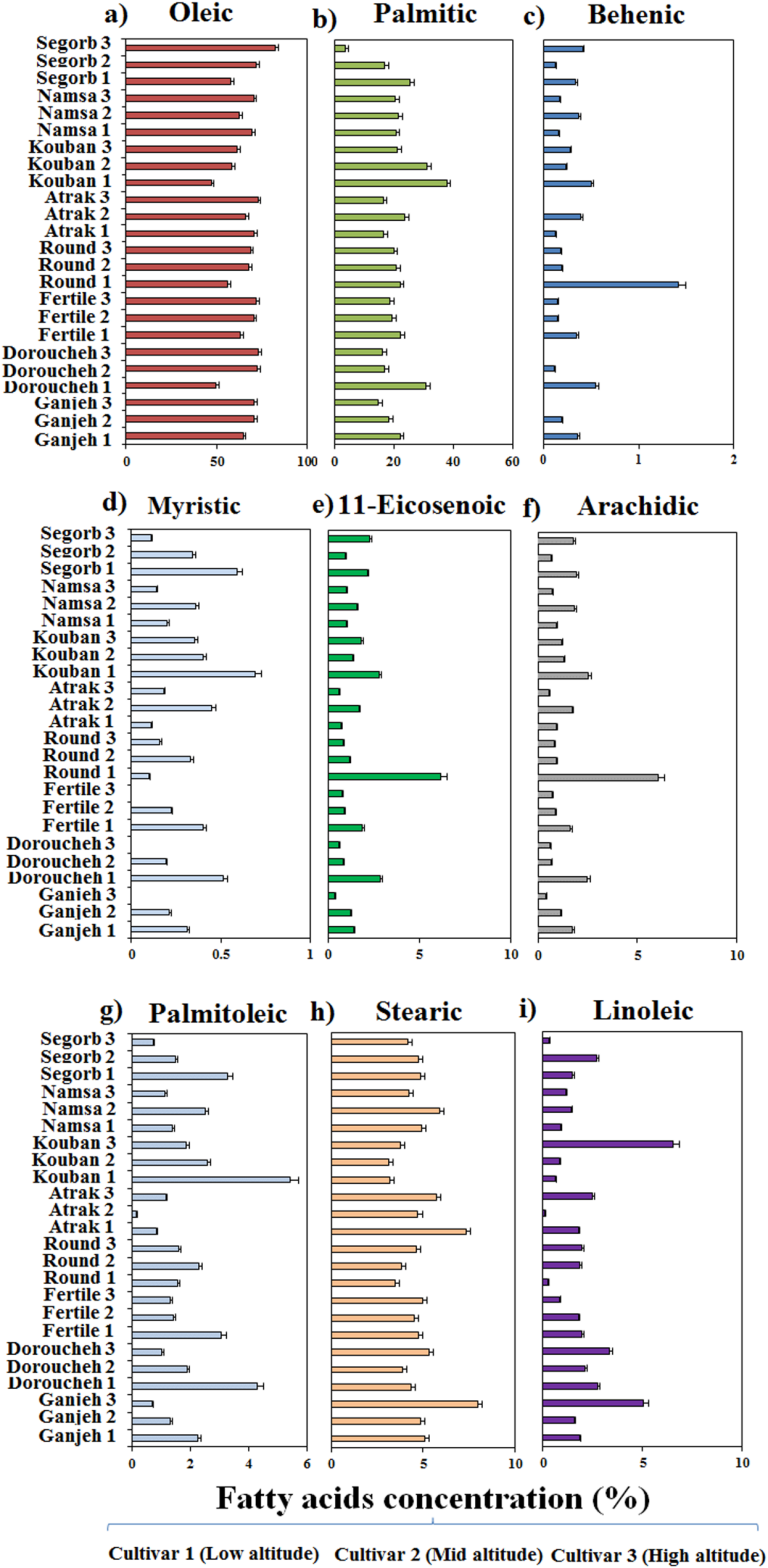
Variations in oleic acid (**a**), palmitic acid (**b**), behenic acid (**c**), myristic acid (**d**), 11-Eicosenoic acid (**e**), arachidic acid (**f**), palmitoleic acid (**g**), stearic acid (**h**), and linoleic acid (**i**) of hazelnut cultivars according to the altitude’s gradients. The numbered target cultivar from 1 to 3 represents the sampled cultivar from low altitude (0-500 m), sampled cultivar from mid altitude (500-1000 m), and sampled cultivar from high altitude (>1000 m), respectively

**Fig. 4:**
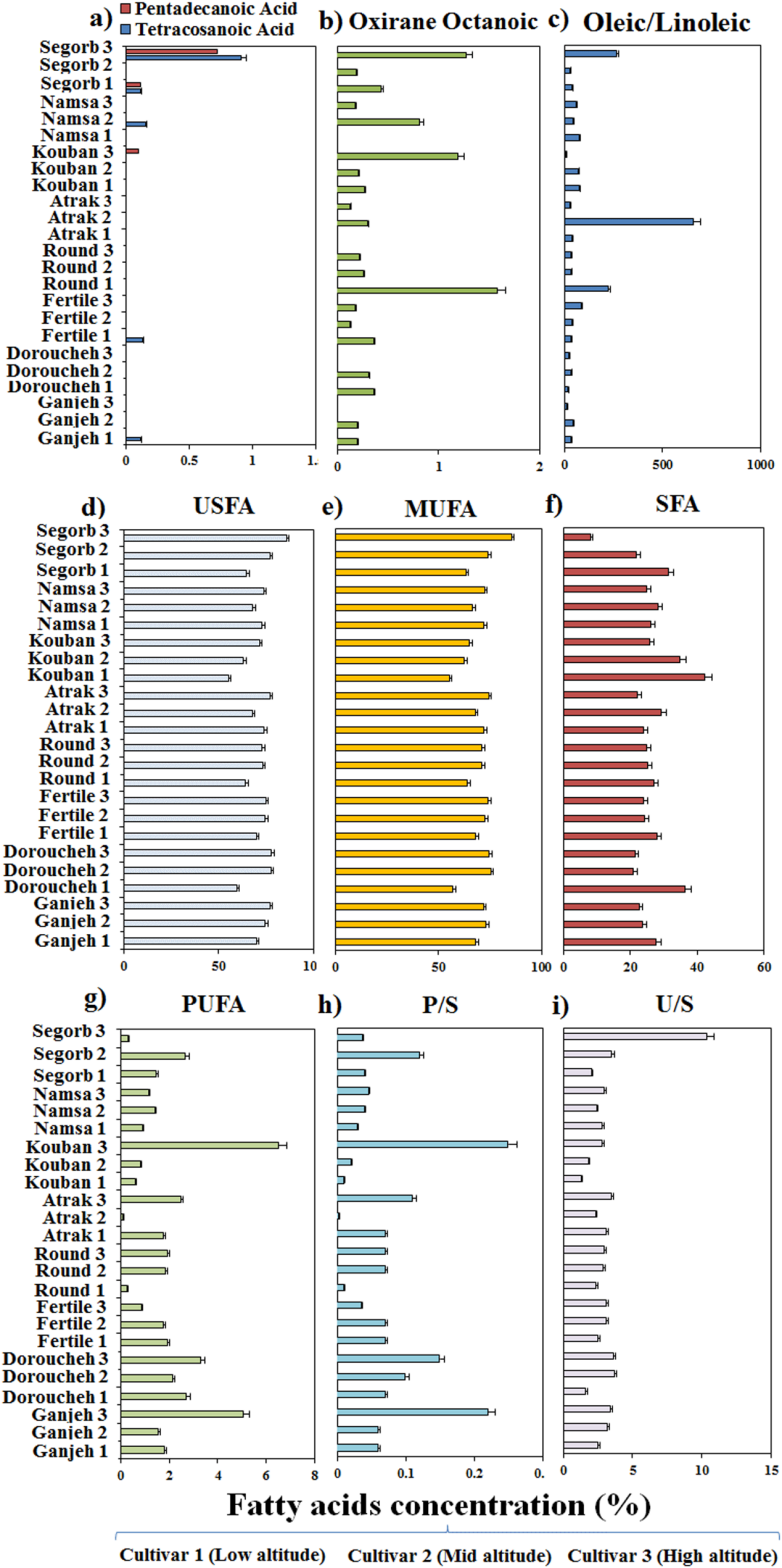
Variations in tetracosanoic and pentadecanoic acids (**a**), oxirane octanoic acid (**b**), oleic/linoleic acid (**c**), USFAs (unsaturated fatty acids) (**d**), MUSAs (monounsaturated fatty acids) (**e**), SFA (saturated fatty acids) (**f**), PUFA (polyunsaturated fatty acids) (**g**), polyunsaturated fatty acids to saturated fatty acids (P/S) (**h**), and unsaturated to saturated fatty acids (U/S) (**i**) of hazelnut cultivars according to the altitude’s gradients. The numbered target cultivar from 1 to 3 represents the sampled cultivar from low altitude (0-500 m), sampled cultivar from mid altitude (500-1000 m), and sampled cultivar from high altitude (>1000 m), respectively

The FA composition of the hazelnut cultivars sampled at three different altitudes was characterized by using the GC-MS technique. The Duncan’s test revealed that the altitudinal gradients had a significant difference (*p* ≤ 0.01) on the FA composition of the hazelnut kernel (Figs. 3 and 4). Among the identified FAs, oleic acid (C18:1) detected as the major component with a higher concentration in the surveyed hazelnut cultivars, which accounted for approximately 63% of the total FA profile. It is noteworthy that oleic acid production was more influenced by cultivar type than by altitude, and therefore all cultivars were able to produce oleic acid. Despite this, as altitude increased, the oleic acid content gradually augmented in all cultivars, except for Namsa and Atrak. However, highest levels of oleic acid had belonged to Segorb (82.37%), Doroucheh (72.92%), and Fertile (71.95%), respectively, which were sampled from high altitude (Fig. 3a). Likewise, palmitic (C16:0) acid ranked in second place, oscillating from 31.61% in the Segorb cultivar to 37.86% in the Kouban cultivar (Fig. 3b). In contrast to oleic acid, the level of palmitic acid was gradually declined with rising altitude. Therefore, the high production of palmitic acid in oils at low altitude was achieved for Kouban and Doroucheh cultivars, individually. Besides this, the Segorb cultivar, with high production of oleic acid indicated lowest level of palmitic acid, suggesting that the palmitic acid synthesis is affected by altitudinal gradients much more than oleic acid synthesis in the oil of surveyed cultivars. The other FAs identified in the cultivar oil were stearic acid (C18:0), linoleic acid (C18:2), and palmitoleic acid (C16:1) in order of abundance. In addition, trace amounts of myristic (C14:0), arachidic (C20:0), behenic (C22:0), 11-Eicosenoic (C20:1), tetracosanoic (C24:0), pentadecanoic (C15:0), and oxirane octanoic acids were also characterized in cultivars oil. Overall, these twelve FAs together accounted more than 99% of the FA profile in the oil samples.

In general, the FA profile was substantially adjusted by all sources of variance (*p* ≤ 0.01), although it was strongly affected by altitude gradient and altitude-cultivar type interaction. Thus, the contribution of altitude to the production of all FAs except for oleic, stearic acids, and oxirane octanoic acid, was much greater than the contribution of the cultivar type. As a result of the Fig. 3, the contents of behenic acid, myristic acid, 11-Eicosenoic acid, arachidic acid, and palmitoleic acid were dramatically declined in the cultivar’s oil with rising the altitude. However, at low altitude (0–500 m), the cultivar contained 1.42% (Round) behenic acid, 0.69% (Kouban) myristic acid, 6.19% (Round) 11-Eicosenoic acid, 6.05% (Round) arachidic acid, and 5.43% (Kouban) palmitoleic acid and thus, they displayed the highest concentration of target FAs in sample oil (Fig. 3c, d, e, f, and g). Conversely, the content of stearic and linoleic acids promoted along with the rise in altitude. Therefore, at high altitude (> 1000 m), the cultivars contained 8% (Ganjeh) and 7.35% (Atrak) stearic acid, and 6.52% (Kouban) linoleic acid (Fig. 3h, and i). Additionally, some special FAs such as tetracosanoic, pentadecanoic, and oxirane octanoic acids were identified in single cultivar, with their contents being strongly affected by altitude-cultivar type interaction. Considerable concentrations of tetracosanoic and pentadecanoic acids were found only in the Segorb cultivar grown at high altitude (Fig. 4a). The maximum content of oxirane octanoic acid was attained for Round at low altitude and for Kouban, and Segorb cultivars at high altitude (Fig. 4b).

While it was absent in some cultivars, this indicates that the production of oxirane octanoic acid in oil sample is strongly dependent on the altitude-cultivar type interaction. This aspect is fully validated by oleic/linoleic acid ratio, which exhibits that the highest ratio of them was obtained for the Atrak cultivar at mid altitude, while the highest amount was obtained for Segorb at high altitude (Fig. 4c). More than 75% of the FA profile of the cultivar’s oil belonged to USFAs (unsaturated fatty acids) and ranged from a mean of 76.88% (Atrak) to 85.72 (Segorb) (Fig. 4d). USFAs were principally composed of MUSAs (monounsaturated fatty acids), fluctuating from 55.16% (Kouban) to 85.41% (Segorb) (Fig. 4e). Both USFAs and MUSAs exhibited changes depending on the altitudinal gradient for all cultivars; however, their concentration was enhanced in cultivar’s oil along with rising altitude. In contrast, the concentration level of SFAs (saturated fatty acids) decreased in oil samples along with rising altitude (Fig. 4f). Thus, the cultivars sampled from low altitudes had the highest concentration of SFAs. Subsequently, Kouban exhibited the best performance for SFAs (42.22%) at low altitude (0–500 m), while Segorb showed the weakest performance at high altitude. The main contributing SFAs for all cultivars included palmitic acid and stearic acid with traces of myristic acid and behenic acid. Accordingly, oil samples derived from Segorb at high altitude and Kouban at low altitude, possessed the highest and lowest content of MUFAs (oleic acid, palmitoleic acid, and 11-Eicosenoic acid) respectively (Fig. 4e). The utmost levels of PUFA (polyunsaturated fatty acids including oleic acid and linolenic acid) were detected in Kouban (6.52%) at high altitude, while the lowest content was found in Atrak (0.1%) at mid altitude (Fig. 4g). The fatty acid profile analysis demonstrated a high ratio of polyunsaturated fatty acids to saturated fatty acids (P/S with a mean of 0.25%) and a high ratio of unsaturated to saturated fatty acids (U/S with a mean of 10.34%) for Kouban and Segorb at high altitude, respectively (Fig. 4h and i).

### PCA and heatmap analysis

**Fig. 5:**
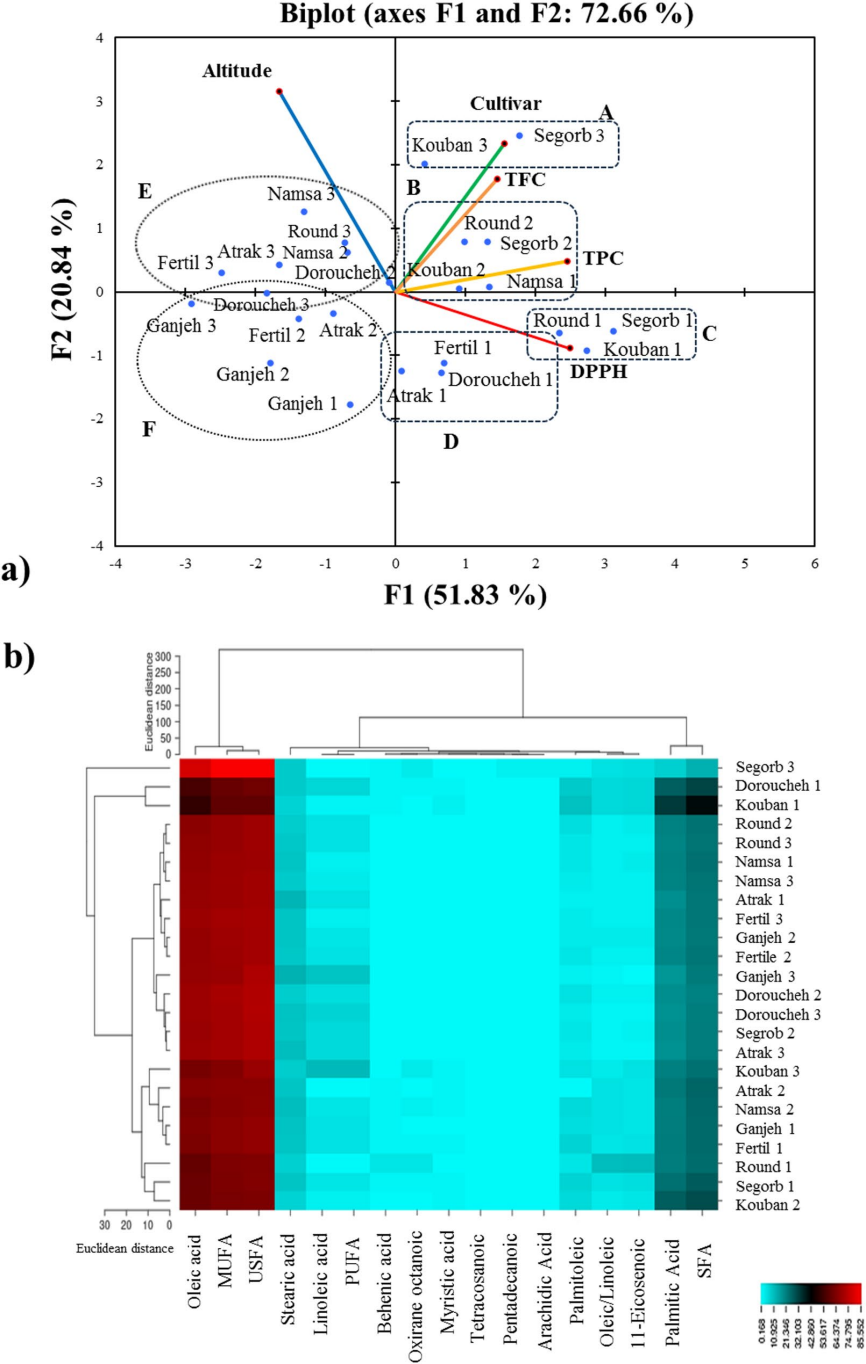
(**a**) Principal component analysis (PCA) based on Euclidean distance performed on antioxidant activity, total phenolic content (TPC), and total flavonoid content (TFC) compounds and (**b**) heat map cluster analysis based on Euclidean distance of fatty acid profile in kernels of the kernel’s hazelnut cultivars according to the altitudinal gradients. (Biplot according to the first two axes). The numbered target cultivar from 1 to 3 represents the sampled cultivar from low altitude (0–500 m), sampled cultivar from mid altitude (500–1000 m), and sampled cultivar from high altitude (> 1000 m), respectively. Cluster heat map. The columns of the heat map represent fatty acids and the rows represent samples. Red color and blue represent the positive correction and negative correction, respectively

**Fig. 6:**
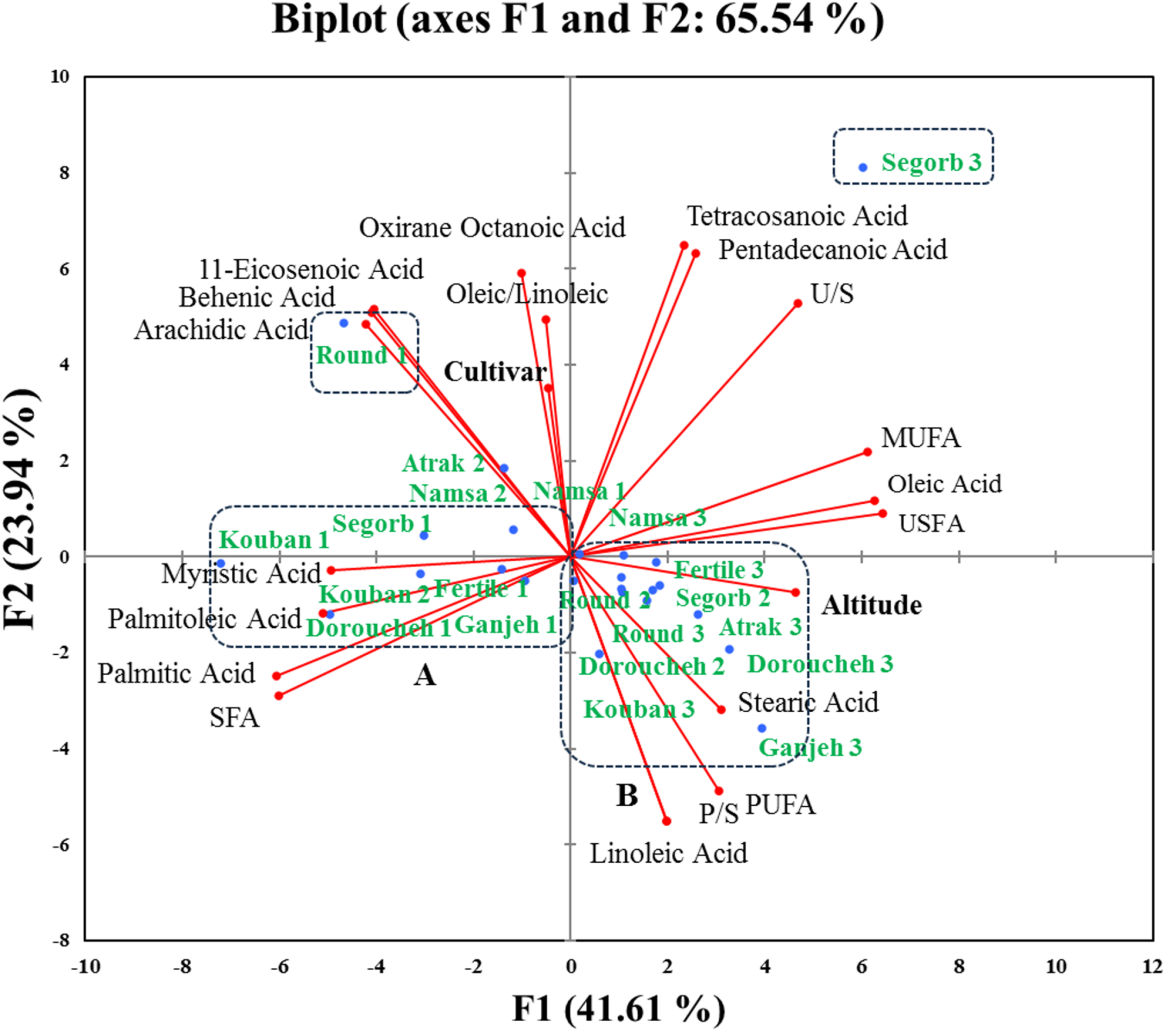
Principal component analysis (PCA) based on Euclidean distance of antioxidant activity, total phenolic content (TPC), and total flavonoid content (TFC) compounds in kernels of the kernel’s hazelnut cultivars according to the altitudinal gradients. (Biplot according to the first two axes). The numbered target cultivar from 1 to 3 represents the sampled cultivar from low altitude (0–500 m), sampled cultivar from mid altitude (500–1000 m), and sampled cultivar from high altitude (> 1000 m), respectively

The PCA analysis conducted to dissect patterns and simplify complexity of data associated with biochemical and FA profiles in hazelnut cultivars, as shown in Figs. 5 and 6. The PCA biplot, conducted for eight cultivars at three altitudes showed a notable alteration in the content of DPPH scavenging activity, TPC, and TFC depending on the cultivar type-altitude interaction, which is accounts for 72.66% of the total variance sources according to PC1 and PC2 (components with eigenvalues ≥ 1.00). It clearly showed that cultivars behaved differently regarding traits affecting the two principal components and their behaviors can be categorized in a clear demarcation manner (Fig. 5a). Hence, most of the cultivar samples from mid and high altitudes (groups E and F) are placed in the 1 st and 3rd quadrants of the PCA plot. This aspect approves that the target cultivars had lowest content for DPPH scavenging activity and TPC, and as a result, the production of these compounds in their kennel’s hazelnuts is less dependent on the mid and high altitudes. In contrast, the cultivars with high antioxidant activity (groups C and D) are placed in 4th quadrant of PCA plot, signifying a strong impact of low altitude-cultivar type on DPPH scavenging activity and TPC content.

Thus, the Segorb, Kouban, and Round scored best cultivars for antioxidant activity and TPC production. Consequently, the mentioned cultivars (groups A and B, respectively) at high altitude demonstrated a high content of TFC, suggesting a strong impact of high altitude-cultivar type on TFC production. The outcome of PCA biplot for FA profile resulted in that modifications in the synthesis of individual FAs in a cultivar’s oil can occur at a prominent altitude depending on cultivar type, accounting for 65.54% of the total variance sources according to PC1 and PC2 (Fig. 6). The PCA biplot indicated that Segorb cultivar sampled from high altitude segregated by high tetracosanoic acid, and pentadecanoic acid contents, as well as U/S, while holding low palmitic acid, palmitoleic acid, myristic acid, and SFA content, which were pooled in the categorized as group A from low altitude, including Ganjeh, kouban, and others. Besides this, Round cultivar sampled from low altitude segregated by high behenic acid, arachidic acid, 11-Eicosenoic acid content. The rise in altitude from mid to high altitudes was prominent in terms of stearic acid, linolenic acid, P/S, and PUFA for cultivars categorized as group B, while the drop in altitude from mid to low altitude was prominent regarding palmitic acid, palmitoleic acid, myristic acid, and SFA for cultivars categorized as group A.

Furthermore, to further approve the links between the FA profile with cultivar type and altitude at different levels, hierarchical cluster analysis based on Euclidean distance method was performed to discover their relationships. As revealed in Fig. 5b, the heatmap analysis categorized cultivars into five clusters according to the FA levels of eight hazelnut cultivars, signifying that altitudinal gradients have indicated different effects on FA concentration. The segregation of Segorb cultivar from high altitude into a distinct group by the PCA biplot was also validated by heatmap analysis as cluster 1, which was specialized due to the high USFA, MUFA, oleic acid, and the appearance of specific acids in their nuts including tetracosanoic and pentadecanoic acids. Likewise, the Kouban and Deroucheh cultivars from low altitude were grouped as cluster 2 due to the high palmitoleic acid, palmitic acid, myristic acid, SFA while constituting low USFA, MUFA, and oleic acid compared other cultivars. Although the low USFA, MUFA, and oleic acid contents in these cultivars were identified by the PCA biplot (Fig. 6), it was not able to more accurately group these two cultivars into one group, as indicated by heatmap (shown as cluster 2 in Fig. 6). Additionally, although the Round cultivar from low altitude was segregated into a distinct group by the PCA, the heatmap analysis grouped this cultivar together with two other cultivars including Segorb from low altitude and Kouban from mid altitude in a cluster (shown as cluster 5 in Fig. 6). Overall, most of the cultivars sampled from three altitudes characterized by relatively same content for USFA, MUFA, and oleic acid. However, the production of some fatty acids in small amounts, such as behenic, arachidic, and oxirane octanoic acids, under the influence of specific altitude conditions led to their grouping into separate clusters by the heatmap analysis. These results confirm that altitude gradient influences the FA production in hazelnut cultivars more than biochemical production.

## Discussion

Salinity, drought (soil water stress), soil fertility, light radiation, chemical exposure, high CO_2_, temperature fluctuations, and gaseous toxins are the major abiotic stresses that significantly limit the survival and growth of plant in both in vitro and *in vivo* manners ‎[14, 47–53]. It has been well documented that the vertical variation of heat and water in the environment regularly influenced by altitudinal gradients, which leads to systematic and significant changes in ecological factors, such as UV radiation, temperature, and water ‎[‎^54^]. Altitude-dependent natural climate gradients enhance the potential characteristics of plant behavior to temporal climate change. However, investigating the effects of altitudinal gradients on the responses of various genotypes of plants under environmental stresses is critical, as it offers a unique opportunity to understand the biochemical changes and secondary metabolite production in plant organelles.

### Antioxidant capacity, phenolic, and flavonoid compounds

In the current study, the effects of altitudinal gradients at three levels on the fatty acid composition, phenolic, flavonoid, and antioxidant activity of hazelnut cultivars from the northern region of Iran were explored. The results highlighted that the shifts in antioxidant activity, TPC content, TFC content, and FA profile were dependent on cultivar type-, altitude-, and altitude-cultivar interaction. The antioxidant activity and TPC compounds of cultivars showed an inverse relationship along with arising the altitude. So, the cultivars sampled at low altitude, Segorb and Kouban from Astara region, were characterized as superior cultivars with the highest concentrations of TPC content and DPPH scavenging activity. Likewise, the positive impact of low altitude on antioxidant activity and TPC content confirmed by PCA analysis, and consequently was categorized into a distinct group based on their altitude-cultivar interaction. These results align with findings from previous studies, which reported that a low-altitude environment promotes the antioxidant activity and phenolic compounds in hazelnuts ‎ [33], walnuts ‎[‎^55^], Rosa ‎[‎^56^], and strawberries ‎[‎^57^]. On the contrary to this, various studies have proved that the antioxidant capacity and phenolic compounds are promoted along with arising altitude in *Elaeagnus angustifolia* ‎[‎^58^], *Taxus wallichiana* ‎ [31], Andean blackberries ‎[‎^59^], and *Vaccinium glaucoalbum* ‎[‎^60^]. These contradictory findings indicate that the altitude-genetic background interaction plays a key role in the alternation of antioxidant capacity and phenolic content in plant’s organelles, as the present study confirms. The aggressive stressors such as low temperature, high light intensity and UV-B radiation, low oxygen, and oxidative stress are primarily experienced by plants in high-altitude environments. Despite these contradictory findings, our findings agree with the study of Nataraj et al. [‎^21^], which showed that the phenolic content in *Artemisia brevifolia* decreases with increasing altitude. The difference in term of TPC content among the three altitudinal gradients signifies that altitude is the key driving force triggering variation. Thus, the unforeseen decline in TPC content might be induced by the highly extreme of environmental conditions these hazelnut cultivars are facing. Compared with other extreme environments, cold temperature stress is the most predominant factor that disturbs the plant growth, thereby plants cope with it by adjusting their growth cycle and expressing of the biochemical and molecular properties ‎[‎^61^]. Previous studies have clearly emphasized that the phenolic concentration and antioxidant capacity decrease by cold temperature stress during cold acclimation process in plants ‎[‎^62^]. At higher altitudes, cold stress induces oxidative stress via generating a considerable levels of reactive oxygen species (ROS) which leads to lipid peroxidation and damage to plants. To combat oxidative stress, the antioxidant enzymes and phenolic compounds of the antioxidant defense system established to scavenge free radicals. Although hazelnut cultivars presented high TPC content and antioxidant activity at all altitudes, the amount of this production was higher at low altitude than at high altitude. As a result of high activity of phenolic and antioxidant enzymes at the highest altitude to combat oxidative stress, suggesting a slight decrease in TPC concentration and DPPH scavenging activity compared to lower altitudes. This aspect has confirmed by Agrawal and Saklani ‎[‎^63^], who investigated the physiological response of *Picrorhiza kurroa* to cold stress under high altitude. On other hand, the low-altitude environment characterized by high temperatures and sunlight exposure, which influence the hazelnut phytochemistry. Investigations have supported that high temperatures followed by full sunlight enhance the phenolic and antioxidant activity, while lower temperatures decline them ‎ [64], which is consistent with our results. It can be inferred that a specific altitude-cultivar type interaction plays a key role in the production of TPC and antioxidant activity in hazelnuts.

Moreover, results showed that hazelnut cultivars exhibited a non-pattern trend for TFC production. Similar findings have attained in previous studies on hazelnuts ‎ [33] and blackberries ‎[‎^59^], where hazelnut cultivars and blackberries exhibited complex behavior for TFC production; thereby the samples collected at higher altitudes indicated higher concentrations depending on cultivar type. Therefore, the highest TFC was obtained for Segorb and Kouban at higher altitude, for Round and Deroucheh at mid altitude, and for Namsa and Fertil cultivars at lower altitude. This result represents the complexity of flavonoid production and validates the impacts of the multiple factors on TFC content in hazelnut composition. Flavonoids compounds protect plants against abiotic stress and also saved them from high UV radiation, limited oxygen availability, and low temperature stresses ‎[‎^62^]. Therefore, the highest content of TFC at mid and high altitudes can be associated with the emergence of cold temperature and high UV radiation. Several studies have proved that the intensification of flavonoid biosynthesis occurs in *Arabidopsis thaliana* ‎[‎^65^], and *Lavandula angustifolia* Mill.‎ [‎^66^], characterized as a potential enhancers of plant resistance to freezing and cold temperature stresses. Overall, it is notable that the altitudinal gradient interacting with cultivar type plays a switching role in determining the phytochemical profiles of hazelnut fruit. In essence, the cultivar-altitude interaction for flavonoids reveals the hierarchy of stress perception and the genetic architecture of the flavonoid pathway in each cultivar. The complex pattern is a signature of each cultivar’s unique investment strategy across the different flavonoid sub-branches in response to the changing altitudinal stress package. Realizing the mechanisms underlying altitude effects can lead to augmenting cultivation practices, choosing superior cultivars for specific altitudes, and encouraging the production of fruits with nutritional properties and health benefits. Hence, the superior biochemical properties of the Segorb, Kouban, Round cultivars hint at a more formidable internal defense network, earmarking them as prime candidates for cultivation in the demanding environment of higher altitudes.

### Fatty acid characterization under altitude–genotype interaction

The fatty acid profile of hazelnut kernels usually plays an important role in the composition of nutritional value and oil stability during storage ‎[‎^67^]. Many studies have pronounced that the altitudinal gradients can alter the fatty acid metabolism of fruits ‎[33, 35, 36, 38, 56, 68]. The FA composition of the hazelnuts was found to vary significantly (*p* ≤ 0.01) with the region of cultivation under three altitudinal gradients. The outcomes of the current study expressed that changes in target fatty acid of FA composition occur at a specific altitude depending on cultivar type. Accordingly, it was approximately detected that palmitic, behenic, stearic, arachidic, 11-Eicosenoic, myristic, palmitoleic acids improved along with dropping in altitude in certain cultivars, namely Round and Kouban, which are superior cultivars at lower altitude (Astara region). In contrast, the oleic and linoleic acids promoted along with arising in altitude in certain cultivars, namely Ganjeh, Kouban, and Segorb, which are superior cultivars at higher altitude (Eshkevarat region). Besides this, a significant concentration of some fatty acids such as pentadecanoic and tetracosanoic acids was only found associated with unique cultivar at a specific altitude, Segorb at higher altitude, while a considerable concertation of the target fatty acids was not produced in other cultivars at any altitudinal gradient. Equally, the different behavior of hazelnut cultivars for the fatty acid production under altitudinal gradients corroborated by PCA analysis, which obviously indicated that the fatty acid metabolism is intensively affected by altitude-cultivar type interaction. Attaining a high concentration of oxirane octanoic acid for Round at lower altitude, Namsa at mid altitude, and Segorb at higher altitude is more indicative of this interaction. It can be inferred that the quantity of fatty acids in hazelnut kernels at lower altitude was greater than higher altitude. The positive effect of lower altitude on FA composition is also validated by other works in hazelnut ‎ [33] and avocado ‎[‎^69^]. Oleic acid, as primary fatty acid with higher concentration, was enhanced at higher altitude, while the palmitic acid, as the second fatty acid, was promoted at lower altitude in across all cultivars. The same trend for the production of oleic acid at higher altitudes has been confirmed by Beyhan et al.‎ [36] and Gülsoy et al.‎ [33] in hazelnut kernels. In these studies, the increased palmitic acid at higher altitudes (>8%) in hazelnuts is at least 9 times less than the concentration of increased palmitic acid at higher altitude (37.86%) in the present study. Also, linoleic acid increased at high altitudes in the hazelnut cultivars, while in the aforementioned studies its amount decreased sharply with increasing altitude ‎ [33, 36]. These findings clearly emphasize that each cultivar can exhibit specific fatty acid variation behavior depending on its genetic background and high-altitude environment.

High-altitude plants are usually exposed to various environmental stresses such as high UV-radiation, low oxygen, oxidative stress, and cold temperatures, while low-altitude plants are more subjected to high light exposure, and heat stresses or high temperatures ‎[‎^61^]. The mechanism altering of fatty acid composition in plants’ resistance to cold temperature through preserving stability and integrity of cell membranes has been extensively explored ‎ [69, 70]. Promoting the metabolism of USFA in membrane lipids to protect of plants against cold stress in low temperature environments has been proven in the *Arabidopsis thaliana* ‎[‎^71^], olive ‎[‎^72^], potato ‎[‎^73^], and hazelnut ‎[‎^74^]. Principally, the content of USFA, MUFA, and PUFA in hazelnut oils sampled from higher altitudes were greater than that in hazelnut oils sampled from lower altitudes. As a result, same increasing trend for oleic acid (as a MUFA) and linoleic acid (as a PUFA) at higher altitudes is consistent with the findings of the above-mentioned studies, which were probably induced in response to the cold temperatures under the high-altitude environment. Heatmap analysis confirms that the highest oleic acid, PUFA, and MUFA contents were obtained for cultivars (Segorb as a superior cultivar) cultivated at high altitudes. Likewise, it is possible that the high UV-B radiation of high-altitude environment stimulates the synthesis of unsaturated fatty acids by damaging cellular lipid structures. The positive effect of UV-B radiation at high altitudes on fatty acid metabolism in *Artemisia argyi* leaves supports this viewpoint [‎^75^]. Considering with low-altitude plants, it has been stated that the accumulation of saturated fatty acids in membrane structures is a critical function in plants exposed to heat stress via stimulating melting temperature and maintaining the fluidity of membranes ‎[‎^71^, ‎‎^76^]. Particularly, results demonstrated that saturated fatty acid, SFA, increased with along decreasing altitudinal gradient, while unsaturated fatty acid (MUFA and PUFA) decreased, corroborating the previously published works ‎[‎‎^77^-‎‎^79^]. At low altitudes, the saturated fatty acids such as palmitic acid, arachidic acid, 11-Eicosenoic acid, and myristic acid were significantly promoted, which were probably induced under high temperature conditions. Triggers in the biosynthesis of palmitic acid in creeping bentgrass ‎[‎‎^78^], behenic acid in rice ‎[‎‎^80^], stearic acid in tall fescue ‎[‎^81^] and Turkish hazelnut ‎ [33], and arachidic acid and 11-Eicosenoic acid in canola ‎[‎‎^82^] under high temperature have been proven, which supports our results. Furthermore, several studies have found that the decrease in unsaturated fatty acid and increase in saturated fatty acids occur under rainfall conditions of low-altitude environments ‎[‎^83^]. Since the amount of rainfall in the Astara region (1345 mm) at low altitude is much higher than in other regions (Rudsar and Eshkevarat at mid and high altitudes), the possibility of saturated fatty acids synthesis is further enhanced in this region.

### Selecting superior cultivars for the horticultural purposes under altitude–genotype interaction

Knowledge of the fatty acid composition and biochemical profile of hazelnut fruit under different environmental conditions can greatly assist in selecting superior cultivars for cultivation, nutritional health, food processing, postharvest life, and plant breeding purposes. The investigation has underscores that a higher oleic/linoleic ratio generally enhances nutritional value, oil stability, and resistance to degradation [26]. Our results confirmed a suitable ratio of the oleic/linoleic for Round at low altitude, Atrak at mid altitude, and Segorb at high altitude. The presence of a lower amounts of linoleic acid, palmitoleic, and linolenic acid in hazelnut cultivars grown at low altitude can reduce the shelf-life during postharvest due to their instability ‎[‎‎^84^]. Hence, the hazelnut cultivars sampled from high altitude can be suitable candidates for long-term storage. Considering human health, unsaturated fatty acids generate the free radicals during oxidation, which can be harmful to human health ‎‎[‎^67^]. In this study, the fruit consumption of hazelnut cultivars (such as the Kouban cultivar) at low altitude can be associated with high nutritional value to human health due to having lower ratios of the U/S, and P/S. Also, the high levels of MUFA and PUFA along with a low level of the SFA, can enhance applicability in food products ‎[‎^85^], as scored by the hazelnut cultivars grown at high altitude, such as Segorb and Kouban according to our results. When hazelnut cultivation is considered for industrial production under cold temperatures, the cultivars with high USFA/SFA ratios from high altitude contribute to a better selection for this purpose. By characterizing the biochemical and beneficial fatty acid composition across various hazelnut cultivars under altitudinal gradients, scientists can potentially develop cultivars with enhanced quality in the future for various purposes.

## Conclusion

In nutshell, findings of the current study suggest that the effects of altitudinal gradients on the fatty acid composition and biochemical properties of hazelnut fruits were altitude-, cultivar type-, and altitude-cultivar type dependent. Generally, cultivars showed a decreasing trend in antioxidant capacity and TPC content along with arising altitude, while both increasing and decreasing trends were detected for TFC content of hazelnut cultivars. This signifies the predominant effect of altitude-cultivar type interaction on the biochemical properties of hazelnut fruit. Additionally, at high altitudes, the most abundance of unsaturated fatty acids such as oleic acid and linoleic acid was remarkably promoted, while the production of saturated fatty acids declined strongly. At low altitude, the concentration of saturated fatty acids, including palmitic acid, behenic acid, stearic acid, arachidic acid, 11-Eicossenoic acid, and myristic acid, enhanced. Beside this, triggers in the biosynthesis of some single acids such as tetracosanoic and pentadecanoic had occurred at specific high altitudes in specific cultivars (Segorb). This aspect was also observed for oxirane octanoic acid production, where it was found that the highest amounts of this acid were obtained for Segorb and Kouban cultivars at high altitude, while the highest amount was obtained for Round cultivar at low altitude. This clearly shows that the changes in fatty acid composition in eight hazelnut cultivars were not universally affected by altitude-cultivar type interaction. Based on the findings, due to high oleic acid and oil stability, Segorb cultivar is recommended for high-altitude regions targeting premium oil production. Kouban cultivar is suitable for low-altitude regions where high antioxidant capacity is desired for functional food production. The conductance of study in a single year, on a small regional scale, and focusing on the study of kernel composition could be limitations of the present study. In order to examine in more detail, the changes of the fatty acid composition and biochemical properties of the hazelnut cultivars across altitudinal gradients, it is recommended that future studies be conducted on a larger regional scale and over several consecutive years. Likewise, if future studies are integrated with the omics approaches for underlying molecular mechanisms, they can yield excellent results.

## Supplementary Information

Supplementary Material 1.

Supplementary Material 2.

## Data Availability

Data will be available on request.
