# Supplementary material for: Impact of altitudinal gradients on biochemical traits and fatty acid profiles of Iranian hazelnuts
Source: BMC Plant Biol. 2025 Dec 1;26:51. doi: 10.1186/s12870-025-07750-w (PMC12797488; doi:10.1186/s12870-025-07750-w)

**Supplementary Table S1.** The ANAOVA variance analysis of biochemical properties of hazelnut cultivars under altitudinal gradients at significant level of p<0.001.


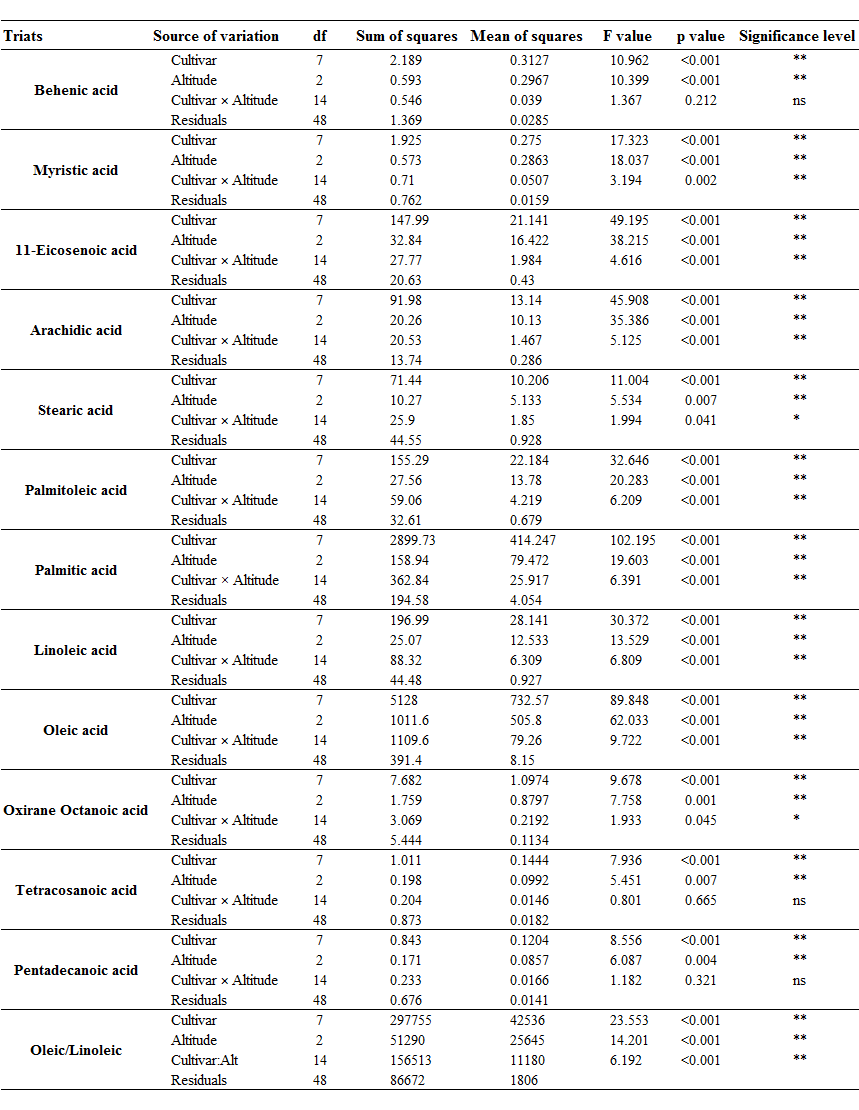


**Supplementary Table S2.** The ANAOVA variance analysis of biochemical properties of hazelnut cultivars under altitudinal gradients at significant level of p<0.001.


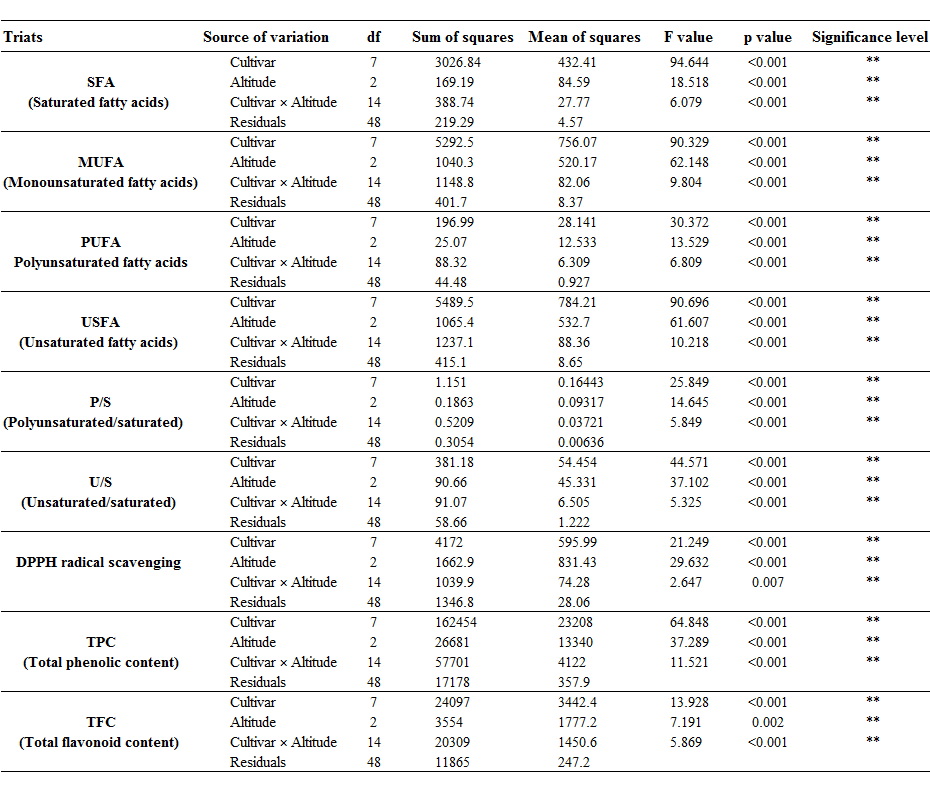

Supplement: Supplementary file 1 — Supplementary Material 1. [file 12870_2025_7750_MOESM1_ESM.docx]
