# Supplementary material for: Impact of altitudinal gradients on biochemical traits and fatty acid profiles of Iranian hazelnuts
Source: BMC Plant Biol. 2025 Dec 1;26:51. doi: 10.1186/s12870-025-07750-w (PMC12797488; doi:10.1186/s12870-025-07750-w)
Supplement: Supplementary file 2 — Supplementary Material 2. [file 12870_2025_7750_MOESM2_ESM.docx]

**
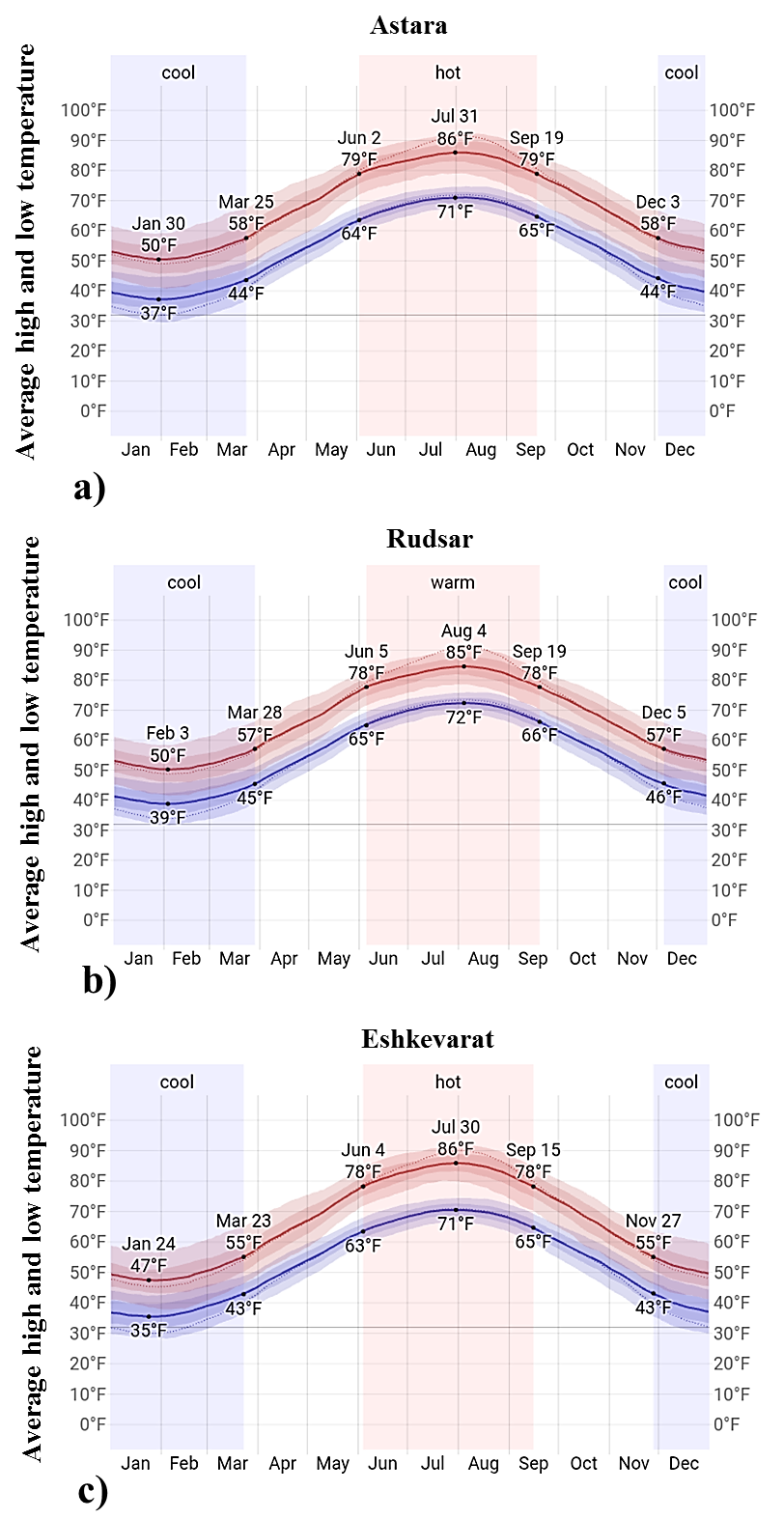
**

**Supplementary Fig. S1.** Average high and low temperature of Astara (a), Rudsar (b), and Eshkevarat (c) for 2024 year, which are located in Gilan province from Iran.


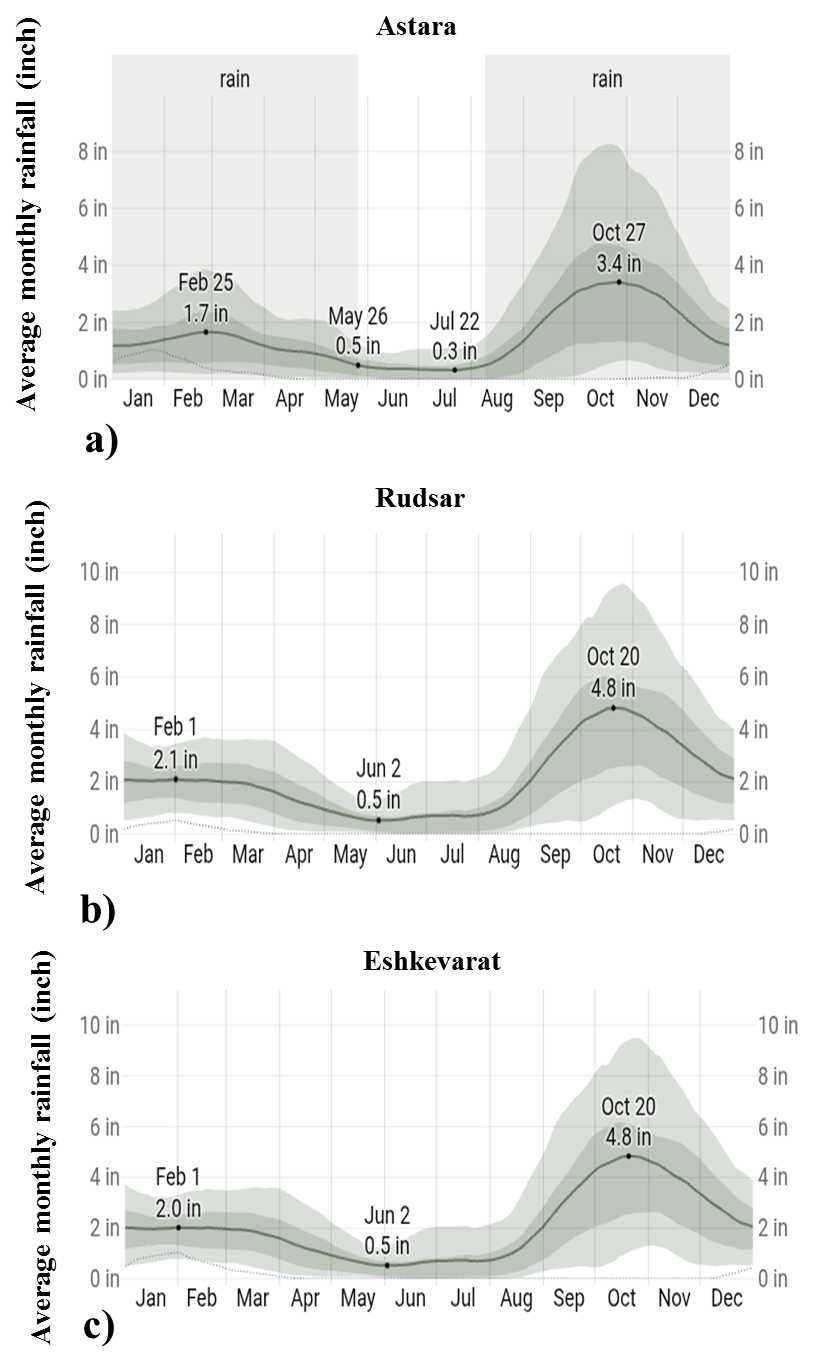


**Supplementary Fig. S2.** Average monthly rainfall of Astara (a), Rudsar (b), and Eshkevarat (c) for 2024 year, which are located in Gilan province from Iran.


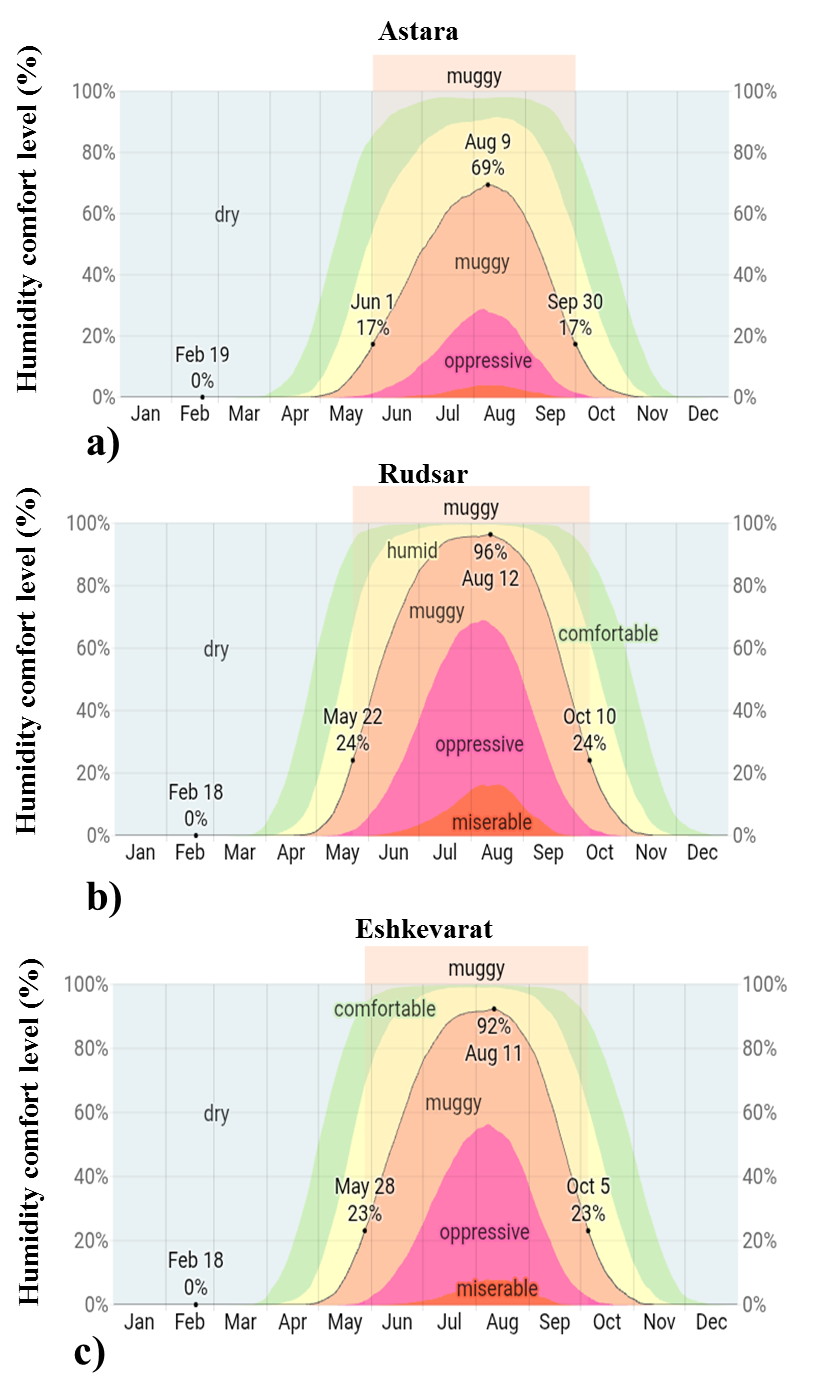


**Supplementary Fig. S3.** Humidity comfort level of Astara (a), Rudsar (b), and Eshkevarat (c) for 2024 year, which are located in Gilan province from Iran.
